# Abnormal repression of *SHP-1*, *SHP-2 and SOCS-1* transcription sustains the activation of the JAK/STAT3 pathway and the progression of the disease in multiple myeloma

**DOI:** 10.1371/journal.pone.0174835

**Published:** 2017-04-03

**Authors:** Asma Beldi-Ferchiou, Nour Skouri, Cyrine Ben Ali, Ines Safra, Abderrahman Abdelkefi, Saloua Ladeb, Karima Mrad, Tarek Ben Othman, Mélika Ben Ahmed

**Affiliations:** 1 Institut Pasteur de Tunis, Laboratory of Clinical Immunology, Tunis, Tunisia; 2 Institut Pasteur de Tunis, Laboratory of Molecular and Cellular Hematology, Tunis, Tunisia; 3 Université de Tunis El Manar, Faculté de Médecine de Tunis, Tunis, Tunisie; 4 Bone Marrow Transplantation Center, Tunis, Tunisia; 5 Salah Azaiez Institute, Department of Pathology, Tunis, Tunisia; Charles P. Darby Children's Research Institute, 173 Ashley Avenue, Charleston, SC 29425, USA, UNITED STATES

## Abstract

Sustained activation of JAK/STAT3 signaling pathway is classically described in Multiple Myeloma (MM). One explanation could be the silencing of the *JAK/STAT* suppressor genes, through the hypermethylation of *SHP-1* and *SOCS-1*, previously demonstrated in MM cell lines or in whole bone marrow aspirates. The link between such suppressor gene silencing and the degree of bone marrow invasion or the treatment response has not been evaluated in depth. Using real-time RT-PCR, we studied the expression profile of three *JAK/STAT* suppressor genes: *SHP-1*, *SHP-2* and *SOCS-1* in plasma cells freshly isolated from the bone marrows of MM patients and healthy controls. Our data demonstrated an abnormal repression of such genes in malignant plasma cells and revealed a significant correlation between such defects and the sustained activation of the *JAK/STAT3* pathway during MM. The repressed expression of *SHP-1* and *SHP-2* correlated significantly with a high initial degree of bone marrow infiltration but was, unexpectedly, associated with a better response to the induction therapy. Collectively, our data provide new evidences that substantiate the contribution of *JAK/STAT* suppressor genes in the pathogenesis of MM. They also highlight the possibility that the decreased gene expression of *SHP-1* and *SHP-2* could be of interest as a new predictive factor of a favorable treatment response, and suggest new potential mechanisms of action of the therapeutic molecules. Whether such defect helps the progression of the disease from monoclonal gammopathy of unknown significance to MM remains, however, to be determined.

## Introduction

Multiple Myeloma (MM) is a hematological malignancy that accounts for about 10% of hematological tumors. It is characterized by plasma cell monoclonal proliferation within the bone marrow, associated with a production of a monoclonal protein [1–3]. Many advances have been made in the field of MM treatment [4]. However, despite the improvement of patient survival achieved by the available therapeutic strategies, MM remains a frequent and an incurable disease [5]. Thus, understanding the biological aberrancies that drive the development of MM is crucial to reduce relapses and to obtain durable remissions.

It is broadly accepted that the bone marrow microenvironment is critical for the development of MM, since it provides the malignant plasma cells with the required cytokines and growth factors [6–8]. Among these cytokines, IL-6 plays fundamental role in the growth and survival of malignant cells, in part through the activation of Janus tyrosine kinases/Signal Transducers and Activators of Transcription (*JAK/STAT*) signaling pathway [9–12]. In MM cells, the *JAK/STAT* signaling pathway seems to be constitutively activated [13–16]. This constitutive activation appears to confer a survival advantage to the MM cells through an anti-apoptotic effect. Besides, it is also involved in MM cell drug-resistance [13, 17].

The *JAK/STAT* pathway is stringently regulated at several steps by 3 main families of proteins: the *PIAS* (Protein Inhibitor of Activated *STATs*), the tyrosine phosphatases (*SHP-1* and *SHP-2*), and the *SOCS* (Suppressor of cytokine signal transduction), particularly *SOCS-1* (reviewed in [18]). Although the *IL-6/JAK/STAT* signaling pathway was largely explored in the field of hematological malignancies, particularly MM, the role played by the *JAK/STAT* suppressor genes was less extensively investigated. Actually, *SHP-1* and *SOCS-1* genes were reported to be highly methylated in MM cell lines or in whole bone marrow aspirates from MM patients [19–22]. Little is known about *SHP-1*, *SHP-2* and *SOCS-1* genes expression within the malignant plasma cells *per se* (freshly isolated from MM patients bone marrows). Moreover, the relationship between the silencing of *JAK/STAT* suppressor genes and the degree of bone marrow invasion or treatment response is not well documented. Herein, we examined the expression of the *JAK/STAT* regulating genes (*SHP-1*, *SHP-2* and *SOCS-1*) in the malignant plasma cells isolated from bone marrows of MM patients. Our data demonstrated an abnormal repression of such genes in freshly isolated malignant plasma cells and revealed a significant correlation between gene silencing of such repressors and the sustained activation of the *JAK/STAT3* pathway in MM. In addition, we demonstrated the presence of a negative correlation between the transcription of the studied genes and the bone marrow plasma cells infiltration degree. In an unexpected manner, repression of *SHP-1*, *SHP-2* genes expression was associated to a better initial treatment response.

## Materials and methods

### Patients and controls

Forty-five patients with newly diagnosed and untreated MM were prospectively enrolled in this study. For each patient, laboratory investigations determined the isotype of the monoclonal immunoglobulin, Durie and Salmon stage as well as the percentage of medullary plasma cell infiltration. After enrollment, all patients received Dexamethasone (DXM) and Thalidomide induction therapy. The response to treatment was evaluated three months later based on the International Myeloma Working Group uniform response criteria for MM [23]. The main biological features of the enrolled patients are summarized in Table 1. The control group consisted of 16 age-and sex-matched bone marrow healthy donors (10 men, 6 women, mean age: 46.3 years). The study protocol was approved by the national medical ethics committee and all patients and healthy donors gave their written informed consent before the study in accordance with the Declaration of Helsinki.

**Table 1:** Demographic and main clinical and biological features of the MM patients at diagnosis.

Patients	n = 45
**Gender**	**27/18**
**Mean age (years)**	**51.7**
**Durie and Salmon stage (n)**	
**stage IIA**	**2**
**stage IIIA**	**36**
**stage IIIB**	**7**
**Mean (range) bone marrow infiltration**	**41% (10–100)**
**Treatment response subcategory (n)** (only 40 patients with an available follow-up data)	
**Complete response**	**9**
**Very good partial response**	**5**
**Partial response**	**21**
**Stable disease**	**5**

### Medullar plasma cell isolation

Bone marrow mononuclear cells were isolated on Ficoll-Hypaque gradient and plasma cells were isolated using CD138 magnetic beads (Miltenyi Biotec, Bergisch-Gladbach, Germany) according to manufacturer’s instructions. Purity was assessed by flow cytometry (FACS Canto, Becton Dickinson) and the following labeled mAbs: FITC-conjugated anti-CD38, PE-Conjugated anti-CD138, PE-Conjugated anti-CD19, Cychrome-Conjugated anti-CD45 and APC-H7-conjougated CD56 (BD Biosciences, Le Pont de Claix, France). Purity of isolated plasma cells (CD38+ CD138+) ranged from 95% to 99%.

### *SOCS-1*, *SHP-1* and *SHP-2* mRNAs expression by Real-Time (RT)-PCR

Total RNA was extracted using RNeasy Mini Kit (Qiagen, Hilden, Germany) according to the manufacturer’s procedure. The quality and purity of the extracted RNA was assessed by a direct visualization on agarose gel electrophoresis and a calculation of the ratio of 260/280 nm absorbance. Extracted RNA was reverse transcribed using the Murine-Moloney Leukemia Virus (MMLV) reverse transcriptase (Invitrogen, Cergy Pontoise, France) and random hexamers (Promega, Madison, WI, USA) according to standard procedures. *SOCS-1*, *SHP-1* and *SHP-2* mRNAs were quantified by real-time RT-PCR using SYBR Green PCR Master Mix (Applied Biosystems, Branchburg, NJ, USA) and 300 nM of the chosen couples of primers. Forward and reverse primers for *SHP-1*, *SHP-2* and *SOCS1* were, respectively, 5’-GGTCACCCACATCAAGGTCAT-3’ and 5’-TGTCGAAGGTCTCCAAACCAC-3’, 5’-GAGAGCAATGACGGCAAGTCT-3’ and 5’-CCTCCACCAACGTCGTATTTC-3’, 5’CCCTTCTTGTAGGATGGTAGCACAC3’ and 5’-GGCTCTGCTGCTGTGGAGAC-3’.

The optimal primers and PCR conditions were previously determined. Accordingly, for each gene, different combinations of primers were tested at three different concentrations using a serial dilution of a positive control. The best couple of primers was chosen based on its sensitivity (the lowest cycle threshold), specificity (a cycle threshold equal to 40 in the non-template controls) and efficiency (slope = -3.3). For each couple of the chosen primers, the PCR product was visualized on agarose gel electrophoresis and a direct sequencing of the PCR product confirmed its specificity. All the samples were tested in duplicate and run on the same plate for the same gene. Amplification was performed using 40 cycles of denaturation at 95°C for 15 s and annealing and extension at 60°C for 1 min using an ABI PRISM 7700 sequence detection system (Applied Biosystems). Data were normalized referring to the expression of the endogenous gene *RPLP0* (*Ribosomal Protein*, *Large*, *P0*) using an available gene expression assay (with a tested efficiency equal to one) and TaqMan PCR Master Mix (Applied Biosystems) by calculating 2^-ΔCT^, with ΔCT the difference in threshold cycles for target and reference.

### Immunochemistry

Immunohistochemistry was performed on paraffin-embedded bone marrow biopsies from 21 patients. Following reduction of endogenous peroxidase activity, deparaffinized slides were incubated at 4°C overnight with a rabbit anti-human phosphorylated *STAT3* (p-STAT3 Tyr705) antibody diluted at 1:50 (clone D3A7; Cell Signaling Technology). Labeling was revealed by the R.T.U. VectaStain universal Elite ABC kit (Vector Laboratories). Briefly, after two washes, slides were incubated with a horse serum blocking solution included in the kit for 20 min. After washing, slides were incubated with a secondary biotinylated antibody, then stained with diaminobenzidine substrate (Novocastra) during 3 min. Slides were then washed twice with distilled water, counterstained with 1% hematoxylin and analyzed under a light microscope at a 40x magnification by two independent investigators in a blinded fashion. Positively stained plasma cells were evaluated for each sample.

### Statistical analysis

Statistical analyses were performed using StatView, SPSS and/or GraphPad Prism software. The levels of mRNA expression for *SHP-1*, *SHP-2* and *SOCS-1* in plasma cells were compared between the different study groups using the non-parametric Mann-Whitney U test. Correlations between the gene expression of *SHP-1*, *SHP-2* and *SOCS-1* and either the bone marrow infiltration, the response to treatment or *STAT3* signaling activation were analyzed using Spearman’s rank correlation. The χ^2^ test was used to compare the frequency of patients exhibiting a hypoexpression of *SOCS-1*, *SHP-1* and *SHP-2* in the different sub-groups identified. The statistical significance was assigned to p values < 0.05.

## Results

### Expression of *SHP-1*, *SHP-2* and *SOCS-1* genes is repressed in malignant plasma cells

**Fig 1:**
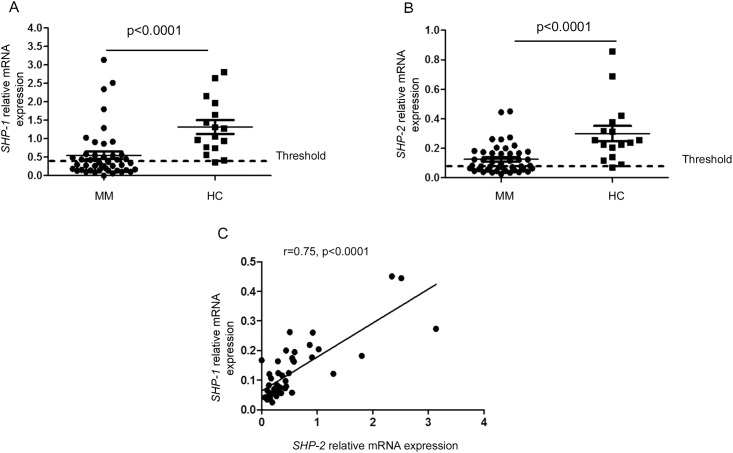
*SHP-1* and *SHP-2* gene expression in bone marrow plasma cells isolated from MM patients and healthy donors. (A, B) Statistical dot plots showing the mRNA levels of *SHP-1* and *SHP-2* relative to the endogenous gene *RPLP0* in sorted bone marrow plasma cells of healthy controls (HC, n = 16) and multiple myeloma patients (MM, n = 45). (C) Correlation between *SHP-1* and *SHP-2* relative gene expression within MM bone marrow plasma cells. Spearman rank correlation (r) and p values are indicated.

The transcription of *SHP-1*, *SHP-2* and *SOCS-1* genes was studied using real-time RT- PCR in the purified bone marrow plasma cells isolated from patients and healthy donors. Within the group of healthy donors, *SHP-1* gene expression in plasma cells ranged from 0.36 to 2.8 with a median of 1.2. Comparatively, *SHP-1* gene expression was significantly reduced within the MM group (p < 0.0001) (Fig 1A). When the threshold of normal transcription of *SHP-1* was calculated as the value of the 5th percentile obtained in the healthy control group, we showed that 27 out of 45 MM patients (60%) displayed a significant reduction in *SHP-1* gene expression (Fig 1A).

Similar results were obtained with *SHP-2*. In the control group, *SHP-2* gene expression ranged from 0.07 to 0.8 with a median of 0.08. Its expression was significantly reduced in the malignant plasma cells from MM patients (p < 0.0001). When the threshold of normal expression of *SHP-2* was determined as above, 23 out of 45 MM patients (51%) had a reduced *SHP-2* gene expression (Fig 1B). Interestingly, in the malignant plasma cells, we found a significant correlation between *SHP-1* and *SHP-2* transcript expression (r = 0.75, p < 0.0001) (Fig 1C).

**Fig 2:**
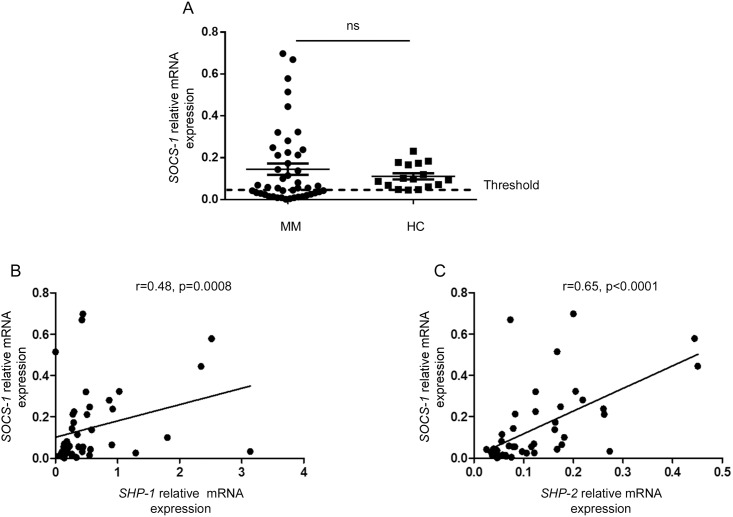
*SOCS-1* gene expression in bone marrow plasma cells isolated from MM patients and healthy donors. (A) Statistical dot plots showing the mRNA levels of *SOCS-1* relative to the endogenous gene *RPLP0* in sorted bone marrow plasma cells of healthy controls (HC, n = 16) and MM patients (MM, n = 45). (B, C) Correlation between *SOCS-1* and *SHP-1*, *SOCS-1* and *SHP-2* relative gene expression in MM bone marrow plasma cells. Spearman rank correlation (r) and p values are indicated.

Regarding *SOCS-1* gene expression, no significant difference was found between the healthy donor and the patient group (Fig 2A). Moreover, within the patient group, *SOCS-1* gene expression profiles were very heterogeneous. Indeed, *SOCS-1* gene expression was increased in 13 MM patients (exceeding the 95th percentile of the healthy control group) and decreased in 20 other cases (below the 5th percentile of the healthy control group). Thus, within the MM group, *SOCS-1* gene expression displayed a different pattern compared to *SHP-1* and *SHP-2*. Nevertheless, *SOCS-1* gene expression was significantly correlated to that of either *SHP-1* or *SHP-2* (Fig 2B and 2C).

### Analysis of *SHP-1*, *SHP-2* and *SOCS-1* gene expression according to the clinicopathological features of MM

#### Bone marrow infiltration

**Fig 3:**
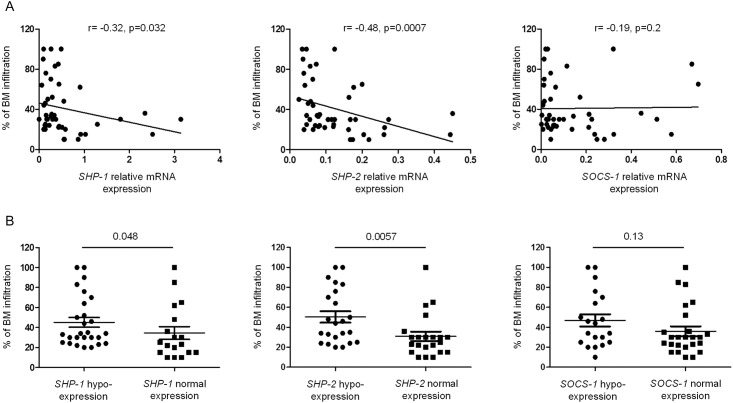
*SHP-1* and *SHP-2* gene expression inversely correlate with the level of bone marrow infiltration. (A) Correlation between bone marrow infiltration and the relative gene expression of *SHP-1*, *SHP-2* and *SOCS-1* in the sorted bone marrow plasma cells. Spearman rank correlation (r) and p values are indicated. (B) The degree of bone marrow infiltration is compared in patients exhibiting normal or decreased expression of *SHP-1*, *SHP-2* and *SOCS-1* genes. P values are indicated.

The percentage of bone marrow infiltration in the patient group varied from 10% to 100%, with a median of 30%. To evaluate *SHP-1*, *SHP-2* and *SOCS-1* gene expression according to the degree of bone marrow infiltration, several statistical analyses were performed. Using Spearman correlation test, we observed the presence of a negative correlation between *SHP-1* and *SHP-2* gene expression and the level of bone marrow infiltration (r = -32, p = 0.032 and r = -48, p = 0.0007, respectively) (Fig 3A). The less *SHP-1* and *SHP-2* were expressed in malignant plasma cells, the more the bone marrow was infiltrated. When the patient group was subdivided into 2 subgroups according to normal or decreased expression of the regulating genes, the level of bone marrow infiltration was significantly increased in the subgroups of patients with lower expression of *SHP-1* or *SHP-2* (p = 0.048 and p = 0.0057, respectively) (Fig 3B).

**Fig 4:**
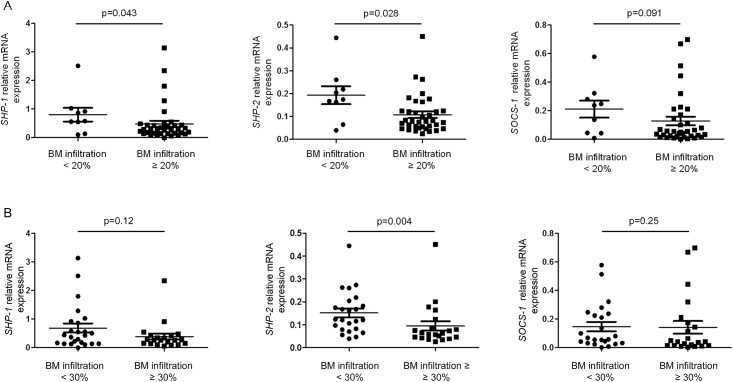
Gene expression of *SHP-1*, *SHP-2* and *SOCS-1* according to the degree of bone marrow infiltration. (A, B) The relative gene expression of *SHP-1*, *SHP-2* and *SOCS-1* was compared in patients subdivided according to two different thresholds (20% and 30%) of bone marrow infiltration. P values are indicated.

The relationship between the expression of regulating genes and the bone marrow infiltration was further analyzed by stratifying patients according to 2 thresholds of infiltration: 20% and 30%. An infiltration ≥ 20% was significantly associated with a reduced expression of *SHP-1* and *SHP-2* genes (p = 0.043 and p = 0.028, respectively) (Fig 4A). Interestingly, an infiltration degree ≥ 30% was associated only with a reduced expression of *SHP-2* (p = 0.004) (Fig 4B).

#### Durie-Salmon staging system

According to the classic Durie-Salmon staging system, the majority of the studied patients (36/45 cases; 80%) exhibited a stage IIIA. Seven patients (15.6%) were classified stage IIIB and only 2 patients (4.4%) displayed a stage IIA. Despite a trend toward decreased expression of the three genes within the stage IIIB subgroup, we could not find any significant difference of expression when compared with stage IIIA and IIIB subgroups (data not shown). Similarly, there was no association between the reduced expression of the studied genes and disease stage (χ^2^ test, p > 0.05) (data not shown).

#### Treatment response

Three months after the beginning of induction therapy, the treatment response was evaluated in 40 out of 45 patients according to the International Myeloma Working Group uniform response criteria [23]. Several analyses were carried out to study the relationship between the expression of the regulating genes and the response to the treatment.

**Fig 5:**
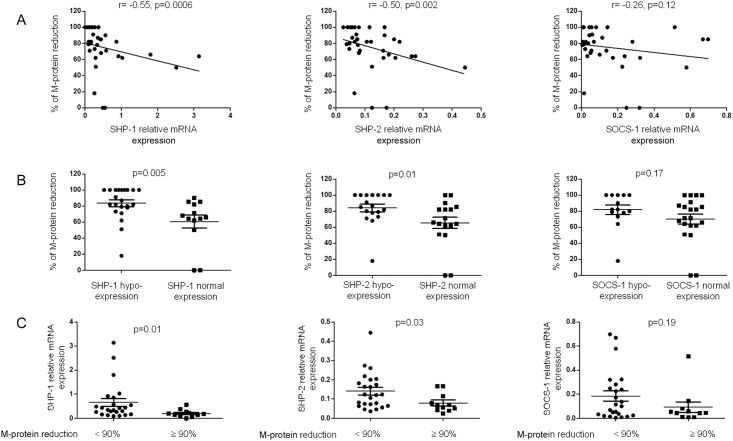
Gene expression of *SHP-1*, *SHP-2* and *SOCS-1* according to the percent reduction of the monoclonal component. (A) Correlation between the reduction of the serum monoclonal component (M-component) and the relative expression of *SHP-1*, *SHP-2* and *SOCS-1* in sorted bone marrow plasma cells. Spearman rank correlation (r) and p values are indicated. (B) Levels of reduction in the serum M-component are compared between patients exhibiting normal or reduced expression of *SHP-1*, *SHP-2* and *SOCS-1*. P values are indicated. (C) The relative gene expression of *SHP-1*, *SHP-2* and *SOCS-1* was compared between the two patient subgroups with a decrease in the M-component <90% or ≥90%. P values are indicated.

Unexpectedly, Spearman correlation analysis showed that the transcription of *SHP-1* and *SHP-2* negatively correlated with the percentage of reduction of the serum monoclonal protein (5 patients with light chain MM were excluded from this analysis) (r = -0.55, p = 0.0006 and r = -0.50, p = 0.002, respectively) (Fig 5A). The less *SHP-1* and *SHP-2* mRNAs were expressed, the stronger the response to the treatment. Additional analyses confirmed this finding. Accordingly, the percent reduction of the serum monoclonal protein was significantly higher within the group of patients exhibiting reduced expression of *SHP-1* or *SHP-2* (p = 0.005 and p = 0.01, respectively) (Fig 5B). When the patients were stratified according to their degree of response to treatment (as assessed by the percent reduction in serum monoclonal protein), *SHP-1* and *SHP-2* gene expression were significantly decreased within the subgroup of patient with a better response (≥ 90% reduction; p = 0.01 and p = 0.03, respectively) (Fig 5B).

Based on the International Myeloma Working Group uniform response criteria [23], we could grossly separate all the 40 patients into “responders” (including patients with either complete or very good partial response), and “non-responders” (including those with partial or no response). Using the χ^2^ test, there was a significant relationship between reduced *SHP-1* expression and the “responder” status (p = 0.026, Table 2).

**Table 2:** Numbers of “non-responders” (patients with partial or no response) and “responders” (patients with either complete or very good partial response) in “reduced expression” or “normal expression” groups for each of the studied gene. P values are indicated.

	Non-responders	Responders	Chi-square test
***SHP-1***			
Reduced expression	13	12	P = 0.026
Normal expression	13	2	
***SHP-2***			
Reduced expression	12	10	P = 0.12
Normal expression	14	4	
***SOCS-1***			
Reduced expression	9	8	P = 0.17
Normal expression	17	6	

**Fig 6:**
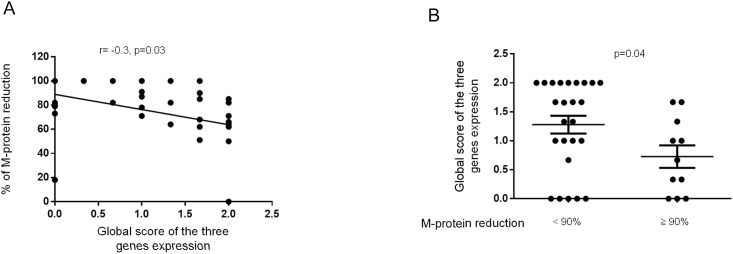
Relationship between treatment response and the global gene expression score. (A) The graph recapitulates the correlation between the global score for *SHP-1*, *SHP-2* and *SOCS-1* gene expression and the percent reduction in the serum monoclonal component (M-component). Spearman rank correlation (r) and p values are indicated. (B) The global expression score of *SHP-1*, *SHP-2* and *SOCS-1* genes was compared between the two patient subgroups with a M-component reduction level <90% or ≥90%. P values are indicated.

Finally, we attributed a score for gene expression ranging from 0 to 2. The score 2 was given to patients presenting a normal expression, 1 for those having a slightly decreased expression but close to the fixed threshold, 0 for an expression below the threshold. A global expression score for the three genes (calculated as the average of the 3 scores attributed to *SHP-1*, *SHP-2* and *SOCS-1* gene expression) was given to each patient. In line with the previous results, this scoring allowed us to show that the global gene expression score negatively correlated with the percent reduction in serum monoclonal protein (r = -0.3, p = 0.03) (Fig 6A). Moreover, this global score was significantly decreased in the subgroup of patients with a better response (≥ 90%; p = 0.04) (Fig 6B).

### *SHP-1*, *SHP-2* and *SOCS-1* gene expression negatively correlates with the activation of the JAK/STAT3 pathway

**Fig 7:**
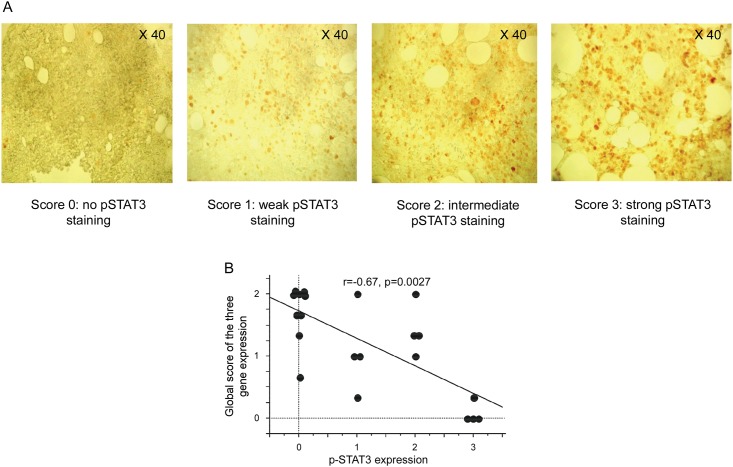
Relationship between the activation of the JAK/STAT pathway and the gene expression of *SHP-1*, *SHP-2* and *SOCS-1*. (A) Consecutive sections of paraffin-embedded MM bone marrow biopsies (40x magnification) were stained with rabbit anti-human p-STAT3 or isotype control. Representative samples are shown from 4 MM patients displaying a score of 0, 1, 2 and 3 (no p-STAT3 staining, weak p-STAT3 staining, intermediate p-STAT3 staining and strong pSTAT3 staining, respectively). (B) The graph recapitulates the correlation between the global score of *SHP-1*, *SHP-2* and *SOCS-1* gene expression and the score for p-STAT3 staining intensity. Spearman rank correlation (r) and p values are indicated.

The JAK/STAT3 activation pathway was analyzed by studying the expression of the phosphorylated form of STAT3 (p-STAT3). Immunohistochemistry was performed on paraffin embedded bone marrow sections from 21 out of 45 enrolled MM patients. A score of STAT3 phosphorylation, ranging from 0 to 3, was attributed according to the intensity of staining (Fig 7A). Activation of the JAK/STAT3 pathway was observed in 12 out of 21 patients (57%) (Table 3). Moreover, in 18 of the 21 patients (85.7%), the p-STAT3 score was concordant with the relative gene expression level *SHP-*1, *SHP-2* and *SOCS-1* (Table 3). Interestingly, a significant correlation was found between the decreased expression of the three studied genes and the activation of the JAK/STAT3 pathway (p = 0.004 for *SHP-1*, p = 0.007 for *SHP-2* and p = 0.02 for *SOCS-1*; data not shown). Finally, the correlation between the activation of the JAK/STAT3 pathway and the global score of *SHP-*1, *SHP-2* and *SOCS-1* gene expression was tested. The global level of expression of these regulating genes was negatively correlated to the activation of the JAK/STAT3 pathway (r = -67, p = 0.0027) (Fig 7B).

**Table 3:** *SHP-1*, *SHP-2* and *SOCS-1* gene expression profiles and the corresponding Immunohistochemistry (IHC) scores for p-STAT3 staining in the indicated patients.

Patient number	SHP-1 relative expression	SHP-2 relative expression	SOCS-1 relative expression	IHC SCORE	Concordance
**1**	normal	normal	normal	0	concordant
**2**	normal	normal	normal	0	concordant
**3**	normal	normal	decreased	0	concordant
**4**	normal	normal	normal	0	concordant
**5**	Slightly decreased	normal	normal	0	concordant
**6**	decreased	decreased	normal	0	discordant
**7**	normal	normal	normal	0	concordant
**8**	normal	normal	normal	0	concordant
**9**	Slightly decreased	normal	normal	0	concordant
**10**	Slightly decreased	Slightly decreased	Slightly decreased	1	concordant
**11**	decreased	decreased	Slightly decreased	1	concordant
**12**	decreased	Slightly decreased	normal	1	concordant
**13**	normal	normal	normal	1	discordant
**14**	decreased	normal	normal	2	concordant
**15**	normal	normal	normal	2	discordant
**16**	decreased	normal	normal	2	concordant
**17**	Slightly decreased	decreased	normal	2	concordant
**18**	decreased	decreased	Slightly decreased	3	concordant
**19**	decreased	decreased	decreased	3	concordant
**20**	decreased	decreased	decreased	3	concordant
**21**	decreased	decreased	decreased	3	concordant

## Discussion

JAK/STAT is a common signaling pathway shared by many cytokines and growth factors [24–28]. This notion supports the important and complex role that this pathway plays in the biology of hematopoietic cells, oncogenesis, and therapy applied to some blood malignancies [28–32]. Particularly, the IL-6R/JAK/STAT3 pathway largely contributes to the MM multistep transformation process and pathogenesis, making its therapeutic targeting an attractive strategy that could favor durable remissions in MM patients [33–36]. The JAK/STAT signaling pathway is regulated at various levels by different molecules such as SOCS-1, SHP-1 and SHP-2, all of which are known to suppress the JAK/STAT signaling pathway. The aberrant and constitutive activation of the JAK/STAT pathway during MM suggests that epigenetic silencing of its regulating genes might be involved in this abnormality. In fact, previous data demonstrated that hypermethylation of *SHP-1* and *SOCS-1* genes is a frequent event in MM. These results were mainly demonstrated in MM cell lines or in whole bone marrow aspirates from MM patients [20–22]. Thus, our first goal was to confirm that the reported hypermethylation resulted in the silencing or at least repression in the expression of these genes within the malignant plasma cells *per se*. Using RT-PCR, we could study the gene expression profile of *SHP-1*, *SHP-2* and *SOCS-1* in plasma cells freshly isolated from the bone marrow of MM patients. Compared with healthy donors, we were able to demonstrate the presence of a global and significant decrease in *SHP-1* and *SHP-2* gene expression in patient plasma cells. Thus, these finding are in line with the reported hypermethylation of *SHP-1* in MM [21]. Moreover, we found a very close and significant correlation between the inactivation of *SHP-1* and *SHP-2* genes, suggesting the involvement of a common mechanism underlying their inactivation during MM. Even though the *SOCS-1* gene expression pattern differs from those of *SHP-1* and *SHP-2*, we could demonstrate that *SOCS-1* gene expression was inactivated in about 44% of patients. This result further corroborates one previous study showing a hypermethylation of *SOCS-1* in 40% of MM patients [22]. The heterogeneous profile of *SOCS-1* gene expression leads us to hypothesize that the mechanisms involved in the inactivation of *SOCS-1* differ from those supporting the inhibition of *SHP-1* and *SHP-2*. Surprisingly, some of our patients (13 out of 45; 29%) exhibited a significantly enhanced expression of the *SOCS-1* gene. *SOCS-1* is classically described as a tumor suppressor in many cancers, including hematopoietic malignancies. However, its ambivalent role was previously reported, since its overexpression seems to be associated to an oncogenic effect in other settings (reviewed in [37]). Moreover, this hyperexpression could also be beneficial by inhibiting the oncogenic events through the degradation of oncoproteins [38]. The limited number of patients with hyper-expression of *SOCS-1* did not allow us to characterize in depth this unexpected feature.

The direct link between the hypermethylation of *SHP-1*, *SHP-2* and *SOCS-1* genes and the hyperactivation of the JAK/STAT signaling pathway in MM is not well established. Indeed, such correlation was only studied in MM cell lines [22]. Notably, using immunohistochemistry to study p-STAT3 on patients’ bone marrow specimens, we observed the presence of a global negative correlation between the sustained activation of the JAK/STAT3 pathway and the gene expression of *SHP-1*, *SHP-2* and *SOCS-1* in patient plasma cells.

Data evaluating the relation between genes regulating the JAK/STAT3 pathway and the bone marrow infiltration status at MM diagnosis is lacking. Thus, in a second step, we studied the relationship between the gene expression of *SHP-1*, *SHP-2* and *SOCS-1* and the degree of patient bone marrow infiltration at baseline. Our analyses highlighted that the inactivation of *SHP-1* and *SHP-2* was significantly associated with a high degree of initial bone marrow invasion. Thus, we consolidate the idea that the inactivation of genes regulating the JAK/STAT3 pathway can support tumor expansion through the repression of JAK/STAT signaling. In addition to its anti-apoptotic effect, the JAK/STAT pathway can also enhance IL-6 production [39, 40]. This can lead to the establishment of a positive feedback loop that amplifies the IL-6R/JAK/STAT3 pathway. It is also likely that the uncontrolled JAK/STAT signaling prompts plasma cells to be more responsive to the survival signals delivered by the microenvironment (e.g. IL-6). Moreover, the significant correlation between bone marrow infiltration and the inhibition of *SHP-1* and *SHP-2* gene expression raises the possibility that their degree of inactivation may represent a biomarker that indirectly reflects tumor mass and the level of bone marrow invasion at diagnosis. Finally, it was suggested that aberrant methylation of some genes, such as *SHP-1* and *SOCS-1*, helps the progression from monoclonal gammopathy of undetermined significance (MGUS) to MM [19, 41]. In line with this, since our results show that the decreased gene expression of *SHP-1*, *SHP-2* was associated with a more extensive bone marrow invasion at diagnosis, we can hypothesize that such patients may be those who progressed more rapidly from MGUS to MM.

The JAK-STAT pathway was incriminated in MM chemo-resistance to multiple agents, including dexamethasone. Indeed, JAK-STAT inhibitors can revert dexamethasone-resistance in MM cells [13, 15, 17, 42–44]. The involvement of JAK-STAT signaling in drug resistance to thalidomide or its analogs is not described. Unexpectedly, our results rather suggest that the inactivation of *SHP-1* and *SHP-2* genes is associated with a strong response to combined dexamethasone/thalidomide induction therapy. Indeed, all the analyses performed showed that the expression of *SHP-1* and *SHP-2* genes inversely correlates with the degree of treatment response. The less *SHP-1* and *SHP-2* genes were expressed, the better the response to treatment. A favorable response was globally more associated to the inactivation of *SHP-1* than to that of *SHP-2*. Accordingly, when patients were subdivided according to the International Myeloma Working Group uniform response criteria into “responders” (complete or very good partial response), and “non-responders” (partial or no response), only *SHP-1* inactivation was significantly associated to the “responder” status. One explanation for the association of a favorable response with the inactivation of *SHP* gene expression could be that therapeutic agents, particularly thalidomide, act through the inhibition of cytokines such as IL-6 that sustain tumor growth and survival [45]. Patients exhibiting a decreased expression of the regulating genes could be therefore more susceptible to the growth factor starvation occurring during treatment. Hence, the level of SHP gene expression could be a predictive factor of a favorable response to induction therapy. Another hypothesis could be that thalidomide and\or dexamethasone may act similarly to other molecules by inducing the transcription of *SHP-1/2* in the malignant plasma cells [46–49]. Subsequently, this could efficiently inhibit the JAK/STAT pathway and induce apoptosis of tumor cells. In this setting, thalidomide and\or dexamethasone would be more effective in patients showing a significant inactivation of SHP gene expression. This could be of interest to adapt and personalize the available therapies according to patient profiles.

Overall, our data provide evidence that repression of *SHP-1*, *SHP-2* and *SOCS-1* gene expression in patient plasma cells supports the constitutive activation of the JAK/STAT3 pathway and probably leads to higher degrees of bone marrow invasion. Interestingly, this aberrancy is associated to a better response to thalidomide and dexamethasone induction therapy. The identification of such subgroups of patients showing greater or lesser responsiveness to particular anti-cancer therapies could be helpful in the development of more adapted treatment strategy. Whether the gene silencing of *SHP-1/2* and *SOCS-1* favors the progression from MGUS to MM remains to be determined. Finally, our work further emphasizes the implication of *SHP-1*, *SHP-2* and *SOCS-1* genes as suppressors of tumor growth in different cancers [37, 50–52].
